# Supplementary material for: The ATP Synthase Subunits FfATPh, FfATP5, and FfATPb Regulate the Development, Pathogenicity, and Fungicide Sensitivity of Fusarium fujikuroi
Source: Int J Mol Sci. 2023 Aug 26;24(17):13273. doi: 10.3390/ijms241713273 (PMC10487771; doi:10.3390/ijms241713273)
Supplement: Supplementary file 1 [file ijms-24-13273-s001.zip › Supplementary Figures.pdf]

Supplementary Figures

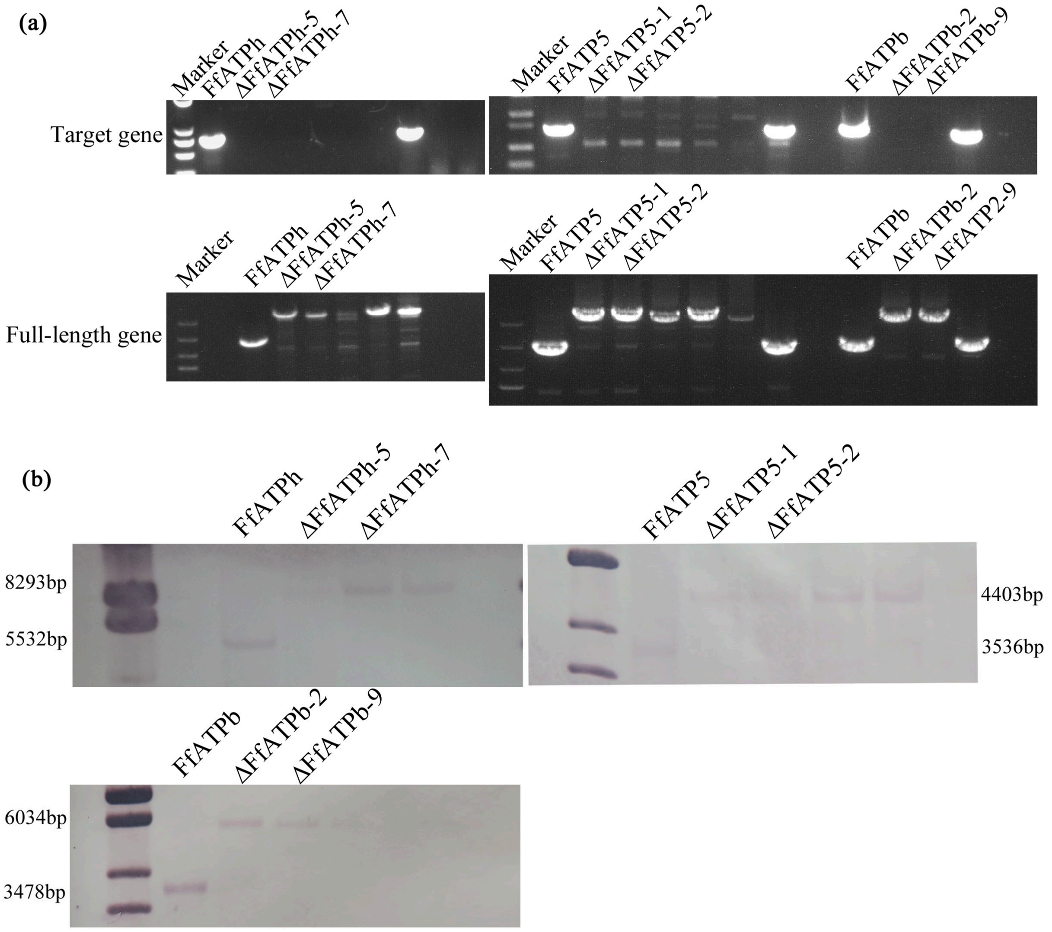

Figure S1. Generation and confirmation of FfATPh, FfATP5, and FfATPb deletion mutants. **(a)** Confirmation of FfATPh, FfATP5, and FfATPb deletion mutants by PCR strategy. Primer pairs FfATPh-inF/FfATPh-inR, FfATP5-inF/FfATP5-inR, and FfATPb-inF/FfATPb-inR were used to verify the target fragment. The PCR amplification cannot validate the DNA bands of the deletion mutants. Primer pairs FfATPh-UF/FfATPh-DR, FfATP5-UF/FfATP5-DR, and FfATPb-UF/FfATPb-DR were used to verify the full-length fragment. Due to the insertion of the HPH-HSV-tk fragment, the ⊿FfATPh DNA fragment is 2761 bp longer than the wild-type DNA fragment, the ⊿FfATP5 DNA fragment is 2245 bp longer than the wild-type DNA fragment, the ⊿FfATPb DNA fragment is 2256 bp longer than the wild-type DNA fragment. **(b)** Southern blot analysis of WT, FfATPh, FfATP5, and FfATPb deletion mutants. Total DNA of WT, ΔFfATPh was digested by HindIII, Probe about 616 bp was located in upstream of FfATPh; Total DNA of WT, ΔFfATP5 was digested by ClaI, Probe about 593 bp was located in downstream of FfATP5; Total DNA of WT, ΔFfATPb was digested by PmlI, Probe about 546

bp was located in downstream of FfATPb;

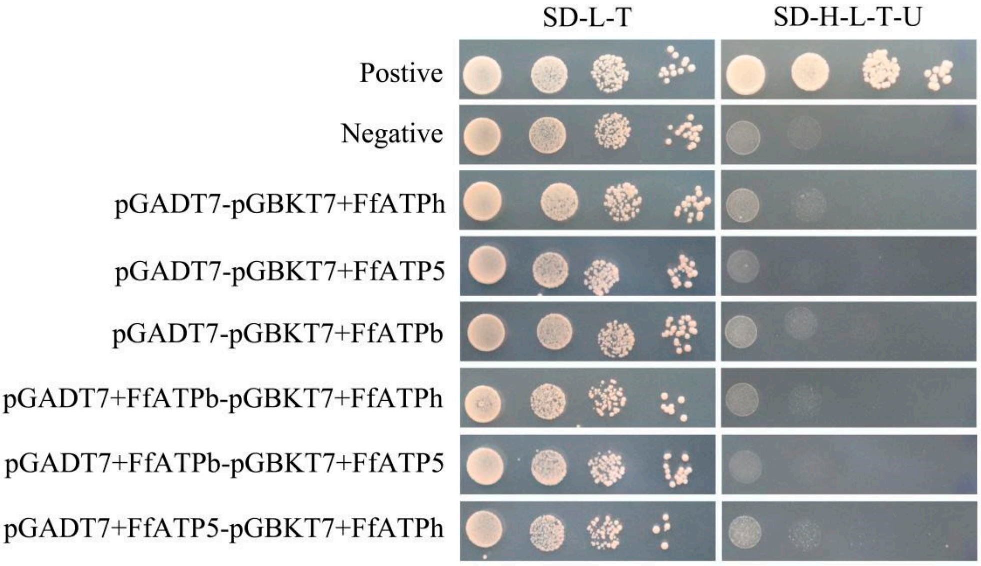

Figure S2. Interaction verification between FfATPb, FfATP5, FfATPh by Yeast two-hybrid.
