# Supplementary material for: The ATP Synthase Subunits FfATPh, FfATP5, and FfATPb Regulate the Development, Pathogenicity, and Fungicide Sensitivity of Fusarium fujikuroi
Source: Int J Mol Sci. 2023 Aug 26;24(17):13273. doi: 10.3390/ijms241713273 (PMC10487771; doi:10.3390/ijms241713273)
Supplement: Supplementary file 1 [file ijms-24-13273-s001.zip › Table S1.pdf]

Table S1 Primers used in this study

Primer	Sequence （5'-3'）	Relevant characteristics
FfATPh-UF	GAGGAGTTGTCAGCCATGTTC	PCR primers to amplify FfATPh upstream fragment
FfATPh-UR	CTTCAATATCATCTTCTGTTTTG AATGGCTGGTGGTG	
FfATPh-DF	AGGAGACAATACCGGAAGGAAC AGCGTTTTTCTTAACCTTTGGG	PCR primers to amplify FfATPh downstream fragment
FfATPh-DR	CATGTGGGTTACGGATGTCT	
FfATPh-inF	TGTTTCTCGTGTCGCCAGA	PCR primers to verify the target fragment of FfATPh
FfATPh-inR	CCTCAAGCCAGTCAGCAA	
FfATPh-csF	ACTGCGTACATCATCGCAT	PCR primers to amplify FfATPh mutant vector
FfATPh-csR	TGCATTGATCGTCGAACTGT	
FfATPh-TZ-F	CAACATTCGATGCAACAACTGT A	PCR primers to amplify FfATPh probe
FfATPh-TZ-R	CGATGGTGCATGTAACCAAGGA	
FfATPh-RP-F	TTTCGTAGGAACCCAATCTTCAA AATGCTCATTCAACTCTCCAGG	Constructed FfATPh-GFP fusion vector
FfATPh-RP-R	GTGAACAGCTCCTCGCCCTTGCT CACGTGGTGAGGCTCATCCTC	
FfATPh-65-F	GCGAATTGGGTACTCAAATTGG TGCACTTGCTATCACATCTTCT	Constructed FfATPh-PHZ65 fusion vector
FfATPh-65-R	GCTCACCATCGTGGCGATGGAG CGGTGGTGAGGCTCATCCTC	
FfATPh-68-F	GCGAATTGGGTACTCAAATTGG TGCACTTGCTATCACATCTTCT	Constructed FfATPh-PHZ68 fusion vector
FfATPh-68-R	GTTCGGGATCTTGCAGGCAGGG CGGTGGTGAGGCTCATCCTC	
SS-F	ACAGAAGATGATATTGAAGGAG C	PCR primers to amplify Double sieve fragment
SS-R	GTTCCTTCCGGTATTGTCTCCT	
FfATPh-NFlag-F	TTTCGTAGGAACCCAATCTTCAA AATGGATTACAAGGATGACGAC GATA	PCR primers to constructe FfATPh-3xflag fusion vector
FfATPh-NFlag-F1	GATTACAAGGATGACGACGATA AGGACTATAAGGACGATGATGA CAAG	
FfATPh-NFlag-F2	GACTATAAGGACGATGATGACA AGGACTACAAAGATGATGACGA TAAA	
FfATPh-NFlag-F3	GACTACAAAGATGATGACGATA AACTCATTCAACTCTCCAGG	
FfATPh-NFlag-R	GTGAACAGCTCCTCGCCCTTGCT	

Primer	Sequence （5’-3’）	Relevant characteristics
FfATP5-UF	CACTTAGTGGTGAGGCTCATCCT C ATCACCGAGTTCTGCTCACC	PCR primers to amplify FfATP5 upstream fragment
FfATP5-UR	CTTCAATATCATCTTCTGTGACG GCGGCTTCTTTGAC	
FfATP5-DF	AGGAGACAATACCGGAAGGAAC AACCACCAACATAGTATAC	PCR primers to amplify FfATP5 downstream fragment
FfATP5-DR	GCCGCCTTCCTCATACTCCT	
FfATP5-inF	GTTGATGGCACTTATGCTAC	PCR primers to verify the target fragment of FfATP5
FfATP5-inR	GATGGTATGGTCTAGACATA	
FfATP5-csF	CAACTCATGTCATGGGTGCA	PCR primers to amplify FfATP5 mutant vector
FfATP5-csR	ACTCTGGTATGCCTCGCATC	
FfATP5-TZ-R	GACTACTTGGATTGCTGGATGA	PCR primers to amplify FfATP5 probe
FfATP5-TZ-R	CTGATTGCTCGCATGATTCATG	
FfATP5-65-F	GCGAATTGGGTACTCAAATTGG GTTTGACCAGCCATGAAATTC	Constructed FfATP5-PHZ65 fusion vector
FfATP5-65-R	GCTCACCATCGTGGCGCAGAGT GTCCGTGAGGAG	
FfATP5-68-F	GCGAATTGGGTACTCAAATTGG GTTTGACCAGCCATGAAATTC	Constructed FfATPh-PHZ68 fusion vector
FfATP5-68-R	GTTCGGGATCTTGCAGGCAGGG CGCAGAGTGTCCGTGAGGAG	
FfATP5-RP-F	TTTCGTAGGAACCCAATCTTCAA AATGTTCTCGCGACAGGTTCT	Constructed FfATP5-GFP fusion vector
FfATP5-RP-R	GTGAACAGCTCCTCGCCCTTGCT CACCAGAGTGTCCGTGAGGAG	
FfATPb-UF	AATCAGAGGCGACTTACTTG	PCR primers to amplify FfATPb upstream fragment
FfATPb-UR	CTTCAATATCATCTTCTGTTGTG GGATGGTTCGTGAT	
FfATPb-DF	AGGAGACAATACCGGAAGGAAC ATAATTAAACGCCGCCGC	PCR primers to amplify FfATPb downstream fragment
FfATPb-DR	AGTACTGGGTAGACTTGCCA	
FfATPb-inF	CTCCTTATATGTAGGCGCTC	PCR primers to verify the target fragment of FfATPb
FfATPb-inR	CTCTCAATATCCGCAACACT	
FfATPb-csF	TGTGTACTCGTCCTTGTCAT	PCR primers to amplify FfATPb mutant vector
FfATPb-csR	AGCAATGTTCGAACCAGTCAG	
FfATPb-TZ-F	GTGTCATAACAACACCTGTGC	PCR primers to amplify FfATPb probe
FfATPb-TZ-R	TCGTCAAGTATCCTGCTTTACG	

Primer	Sequence （5’-3’）	Relevant characteristics
FfATPb-RP-F	TTTCGTAGGAACCCAATCTTCAA AATGGCGTCACGCTTGGTAC	Constructed FfATPb-GFP fusion vector
FfATPb-RP-R	GTGAACAGCTCCTCGCCCTTGCT CACTTGGGCCTTGGAGGCAAC	
FfATPb-Flag-ZS-F	GGCGAATTGGGTACTCAAATTG GTTGATGTTGTAGGAATGCTTCG TG	PCR primers to constructe FfATPb-3xflag fusion vector
FfATPb-3xflag-UR1	CTTATCGTCGTCATCCTTGTAAT CTTGGGCCTTGGAGGCAACGAT C	
FfATPb-3xflag-UR2	CTTGTCATCATCGTCCTTATAGT CCTTATCGTCGTCATCCTTGTAA TC	
FfATPb-3xflag-UR3	CTATTTATCGTCATCATCTTTGT AGTCCTTGTCATCATCGTCCTTA TAG	
FfATPb-3xflag-UR4	ACAGCTCCTCGCCCTTGCTC ACCTATTTATCGTCATCATCTTT G	
FfATPb-56-F	GCGAATTGGGTACTCAAATTGG GATGTTGTAGGAATGCTTCGTG	Constructed FfATPb-PHZ65 fusion vector
FfATPb-65-R	GCTCACCATCGTGGCGTTGGGCC TTGGAGGCAAC	
FfATPb-56-F	GCGAATTGGGTACTCAAATTGG GATGTTGTAGGAATGCTTCGTG	Constructed FfATPb-PHZ68 fusion vector
FfATPb-68-R	GTTCGGGATCTTGCAGGCAGGG CGTTGGGCCTTGGAGGCAAC	
